# Cytotoxic effects and comparative analysis of Ni ion uptake by osteoarthritic and physiological osteoblasts

**DOI:** 10.1038/s41598-024-67157-9

**Published:** 2024-07-12

**Authors:** Polina Navratilova, Marketa Vejvodova, Tomas Vaculovic, Iva Slaninova, Jan Emmer, Tomas Tomas, Ludek Ryba, Jan Burda, Monika Pavkova Goldbergova

**Affiliations:** 1 Department of Pathophysiology, Faculty of Medicine, Masaryk University, Kamenice 5, Brno, Czech Republic; 2 Department of Chemistry, Faculty of Science, Masaryk University, Kamenice 5, Brno, Czech Republic; 3 Department of Biology, Faculty of Medicine, Masaryk University, Kamenice 5, Building A6, 62500 Brno, Czech Republic; 4 1st Department of Orthopaedics, St. Anne`S University Hospital, Pekarska 53, Brno, Czech Republic; 5 Department of Orthopaedic Surgery, University Hospital, Jihlavska 20, Brno, Czech Republic

**Keywords:** Nickel, Implant debris, Osteoblasts, Metal uptake, Metal distribution, Laser ablation, Cell biology, Health care, Materials science

## Abstract

Nickel(Ni)-containing materials have been widely used in a wide range of medical applications, including orthopaedics. Despite their excellent properties, there is still a problem with the release of nickel ions into the patient’s body, which can cause changes in the behaviour of surrounding cells and tissues. This study aims to evaluate the effects of Ni on bone cells with an emphasis on the determination of Ni localization in cellular compartments in time. For these purposes, one of the most suitable models for studying the effects induced by metal implants was used—the patient’s osteoarthritic cells. Thanks to this it was possible to simulate the pathophysiological conditions in the patient’s body, as well as to evaluate the response of the cells which come into direct contact with the material after the implantation of the joint replacement. The largest differences in cell viability, proliferation and cell cycle changes occurred between Ni 0.5 mM and 1 mM concentrations. Time-dependent localization of Ni in cells showed that there is a continuous transport of Ni ions between the nucleus and the cytoplasm, as well as between the cell and the environment. Moreover, osteoarthritic osteoblasts showed faster changes in concentration and ability to accumulate more Ni, especially in the nucleus, than physiological osteoblasts. The differences in Ni accumulation process explains the higher sensitivity of patient osteoblasts to Ni and may be crucial in further studies of implant-derived cytotoxic effects.

## Introduction

Nickel (Ni) is a metallic element that can be encountered both in the environment (water, soil and food contaminants), and also in industrial products (clothing fasteners, jewellery, keys, galvanic cells (Ni–Cd batteries)) etc^1,2^. Due to its properties such as corrosion resistance and long-term stability, Ni itself, as well as Ni-containing alloys, is also widely used in medicine^3^, e.g. dental and orthopaedic implants, stents, cardiac pacemakers and other devices. Also some surgical instruments are coated with nickel.

From the point of view of implantology, this is a quite disputable metal. Despite its excellent properties, nickel is known as one of the most “problematic” metals regarding the cytotoxicity, which can lead to hypersensitivity reaction. Hypersensitivity to Ni in the European general population occurs in 8–19% of individuals, which makes Ni the most widespread metal allergen^4^. The crucial role in the Ni effect on cells and/or tissues have among others the nickel ions (primarily Ni^2+^) released both during the implantation process itself, and as a result of mechanical wear or in case of an implant defect, when the surface oxide layer is disturbed^5^. Ni^2+^ ions are able to affect the surrounding cells and tissues, and also get into the circulation, thereby causing not only local, but also systemic inflammation^6^. Discussing the local toxicity of the released nickel ions, it is mainly associated with the accumulation of Ni in the cells that are in direct contact with the implant—in case of bone implants, the osteoblasts/osteocytes. Previous studies describing the effects of Ni on these cells in vitro, noted the cytotoxic effects leading to apoptosis in MLO-Y_4_ osteocytes^7^, as well as the reduction of metabolic activity in osteoblast-like cells^8^. The effect of Ni on the bone quality and potential complications after implantation with respect to bone remodelling was mentioned by Jonitz-Heincke et al. describing a reduced production of collagen 1 and osteoprotegerin (OPG) in primary osteoblasts, and simultaneously an increase in the synthesis of receptor activator of NF-κB ligand (RANKL), interleukin-6 (IL-6) and interleukin-8 (IL-8), which led to the promotion of osteoclastic activity^9^.

Although the cytotoxicity of nickel ions is well described, there is only a limited number of studies that have addressed the issue of ion accumulation in bone cells. Two really important questions regarding the nickel-induced cytotoxicity arise and remain opened—(1) the cellular Ni^2+^ ions accumulation in time with the compartmental effect, and (2) the ratio between nuclear and cytoplasmic Ni concentration.

The aim of this work is to study the effects of Ni^2+^ ions (the designation “Ni” is used in the text) on bone cells. The study is focused on primary osteoblasts isolated from the bone tissue of patients with osteoarthritis (OB-OA) indicated to hip endoprosthesis, which are used to simulate the pathophysiological environment before implant application. Cell line of human osteoblasts (HOB) were used to compare these effects in terms of changes of cell morphology, proliferation and Ni distribution in the cell, as a model of physiological conditions. In this work, concentrations in the range of 0.5–2 mM were tested in order to determine a concentration-dependent level of cytotoxicity to bone cells, as well as their impact on the cell cycle and cell proliferation. Great emphasis was placed on the imaging of Ni ions and monitoring the penetration into cells, as well as Ni distribution and accumulation in the cell (nucleus vs. cytoplasm). The results of these tests were subsequently analysed in terms of differences between physiological osteoblasts and pathophysiological OB-OA cells. While high concentrations may not directly mimic clinical scenarios, they serve important purposes in initial toxicity screening and risk assessment, such as maximum effect determination to helps in understanding the potential toxicity and establishing a baseline for comparison with lower concentrations.

## Materials and methods

### Clinical samples

Patient osteoarthritic osteoblasts (OB-OA) were isolated from bone fragments after total hip replacement surgery. Patients’ bone fragments were obtained thanks to cooperation with the St. Anne’s University Hospital (Brno, Czech Republic) and the University Hospital Brno Bohunice (Brno, Czech Republic). The collection of patient samples was approved by the Ethics Committees of Masaryk University (Brno, Czech Republic) and the two cooperating workplaces. Each patient participating in the study signed an informed consent. The study was designed under the Declaration of Helsinki. The primocultures of osteoblasts were isolated from three osteoarthritic patients and used for further analyses.

### Cell culture

In this study, patient osteoarthritic primary osteoblasts (OB-OA) and commercial cell line (HOB, 406-05A, Cell Applications, Inc.), isolated from adult human bones without pathological affection (declared by provider as normal), were used. The primary OB-OA (N = 3) were isolated from bone fragments according to commonly used protocol for osteoblast isolation^10^. The primary osteoblasts from osteoarthritic subchondral bone exhibited an activated phenotype, characterized by the production of IL-6, MMPs, and adhesive factors, which was assessed through mRNA expression analysis (data not shown). The phenotype of patient primary osteoblasts was also in consensus with data obtained for commercially available Human Osteoblasts-Osteoarthritis (HOb-OA, 406OA-05A) from Cell Applications, Inc. (data not shown). To make the study closest to applicability on the patient cohort, we preferred to use the patients primary cells. Both, primary osteoblasts and the cell line, were cultivated in Dulbecco’s Modified Eagle’s medium/Ham’s nutrient mixture F12 (DMEM/F-12, GE Healthcare) supplemented with 10% FBS (foetal bovine serum, Sigma-Aldrich Co., St. Louis, USA) and 10 mg/ml penicillin–streptomycin (Sigma-Aldrich Co., St. Louis, USA) under standard growth conditions (37 °C, humidified atmosphere, 5% CO_2_). These conditions were maintained throughout the study. Cells at passages 4–6 were used in this study.

### Live-cell imaging using IncuCyte

Monitoring of cell growth and control of cell morphology was performed using the live-cell monitoring system IncuCyte^®^ (S3, Sartorius, Schaarbeek, Belgium). Cells (HOB and OB-OA) were seeded at a density of 1.5 × 10^4^ cells per well in a 96-well plate (TPP 96-well TC Flat Bottom). After the cell attachment (24 h), a solution of nickel(II) chloride hexahydrate (NiCl_2_ · 6H_2_O, Sigma-Aldrich Co., St. Louis, USA) with different concentrations was added. Cells without added NiCl_2_ were used as a control. Scanning was performed every 60 min.

### Analysis of Ni cytotoxicity

Cytotoxic effects of Ni were analysed using Live/Dead^™^ Cell Imaging Kit (ThermoFisher, MA, USA). OB-OA cells were cultured as mentioned above. NiCl_2_ · 6H_2_O solution at concentrations of 0.25 mM, 0.5 mM, 1 mM and 2 mM was added. Cells were cultured for 24 h, after that live/dead labelling was performed according to the manufacturer’s protocol. Fluorescence signals were measured using IncuCyte^®^ Red (dead cells, ex/em 570 nm/602 nm) and Green (live cells, ex/em 488 nm/515 nm) imaging channels. Analysis was performed in triplicate, with three images analysed from each sample. The results were expressed as total green/red object area (µm^2^/Image).

### Flow cytometry analysis of cell cycle

For cell cycle analysis, OB-OA were seeded on a 6-well plate at a density of 2 × 10^4^ cells per well and loaded with 0.5 mM and 1 mM NiCl_2_ as described above. After 24 h of cultivation with Ni, cells were harvested using trypsin and centrifuged at 2500 rpm/5 min/20 °C. The cells were washed with 500 µl of phosphate buffer saline (PBS) and centrifuged again. The pellet was then resuspended in 265 µl of PBS and 735 µl of ice-cold 95% EtOH was added. The samples were incubated for 30 min on ice and subsequently washed three times with PBS. After resuspending the pellet in 250 µl of PBS, 50 µl of 0.1 mg/ml RNase was added, and the samples were incubated for 30 min at 37 °C. Finally, 10 µl of 1 mg/ml propidium iodide was added (incubation for 10 min, 20 °C). The prepared samples were kept on ice (in the dark) until measured.

Cell cycle was analysed on CytoFlex flow cytometry systems (Beckman Coulter, Inc., Brea, CA, USA) using an excitation wavelength of 488 nm and an emission wavelength of 610 nm; B610). Each data sample was saved as a separate flow cytometry standard file (FCS) and analysed using CytExpert software (Beckman Coulter, Inc.).

### Measurement of cell proliferation

Analysis of cell proliferation was performed using resazurin test. Measurements were made for both cell lines after 24 h, 48 h and 72 h of cultivation with or without (control samples) the addition of 0.5 mM and 1 mM Ni. Cell morphology was checked using IncuCyte^®^. After the cell imaging, cell culture medium was removed, and the cells were washed twice with 200 µl of phosphate buffer saline (PBS). Subsequently, 200 µl of DMEM/F-12 containing 3 µg/ml of AlamarBlue^®^ reagent was added to the cells. Samples were incubated for 1 h at 37 °C in the dark. Total fluorescence was measured using a Cytation 3 Cell Imaging Multi-Mode Reader (Agilent Technologies, Inc., CA, USA), with the excitation wavelength set at 560 nm and emission—at 590 nm. A sample of DMEM/F-12 with 3 µg/ml AlamarBlue^®^ (without cells) was used as a blank.

### Ni localisation by laser ablation inductively coupled plasma mass spectrometry

A laser ablation inductively coupled plasma mass spectrometry (LA-ICP-MS) technique was used to study the distribution of Ni in cells. For this experiment, HOB and OB-OA cells were seeded on a culture slide (Ø18 mm) at a density of 3 × 10^4^ cells per slide. The slides were then placed in the wells of 12-well culture plates and the cells were cultured in 1 ml of culture medium. Samples loaded with Ni were prepared as described above. After 24 h of cultivation, the culture medium was aspirated and the cells were washed twice with PBS. Samples were fixed in 1 ml of ice-cold pure methanol for 10 min and then dried. Fixed samples were stored at – 20 °C.

Determination of elemental distribution within the individual cells was performed using LA-ICP-MS set-up consisting of a laser ablation system LSX-213 G2 + (CETAC Technologies) operating with a wavelength of 213 nm coupled to quadrupole ICP-MS spectrometer Agilent 7900 (Agilent Technologies, Japan). The samples on the glass slides were placed in a two-volume ablation cell Helex II with flow rates of 0.6 l min^−1^ and 0.3 l min^−1^. Laser ablation sampling was performed with line-by-line scan mode, using 7 µm laser beam diameter, spacing of 7 µm, scan speed 14 µm·s^−1^, repetition rate 20 Hz and laser beam fluence 3 J·cm^−2^. Before entering ICP-MS, argon (1.0 l·min^−1^) was admixed into helium flow with ablated material. Signals of elements of interest were monitored through the following isotopes with integration times: ^31^P 0.1680 s, ^60^Ni 0.1630 s; with the addition of ^66^Zn (0.1630 s), the total integration time was 0.5000 s. Obtained LA-ICP-MS data was processed into the form of distribution maps of individual isotopes representing elements of interest using lab-made software Ilaps which was created in Python^11^.

According to the formula $TV={\overline{x} }_{BG} +3\cdot {SD}_{BG}$ the threshold values of ^31^P and ^60^Ni were calculated from the intensity matrices obtained from the Ilaps software. These threshold intensities were used to clearly define the cell area (based on ^60^Ni intensities) and their nuclei (based on ^31^P intensities). The treshold value was calculated also for control samples; the value detected was 32.18260232 cps. All intensities lower than these calculated threshold values were replaced by zero in the intensity matrices for each isotope. The ratio of nickel in the nucleus and cytoplasm (N/C ratio) was calculated from values obtained in the intensity matrix of nickel. The cells were identified using nickel and phosphorus intensities—enriched Ni is present in the whole cell (cytoplasm and nucleus) and P is observable in nucleus only. The N/C ratio was calculated from 20 cells in each sample and the values were averaged. Distribution maps had a total of 30,000 pixels, the size of one pixel was 7 × 7 µm.

### Statistical analysis

Statistical processing of the results was performed using GraphPad Prism 9.0.0. analyses tools. The data values are represented as mean ± standard deviation. For statistical analyses of cytotoxicity, cell characteristics and Ni distribution, the non-parametric Kruskal–Wallis test was used. All the measurements were performed at least in triplicate.

### Ethical approval and consent to participate

In this study, the whole blood samples obtained from osteoarthritic patients were used. This study was approved by the Ethics Committee of the Faculty of Medicine of Masaryk University (Brno, Czech Republic), the Ethics Committee of the St. Anne’s University Hospital (Brno, Czech Republic) and the Ethics Committee of the University Hospital Brno Bohunice (Brno, Czech Republic). Each patient signed an informed consent to participate in this study.

## Results

### Ni cytotoxicity

**Figure 1:**
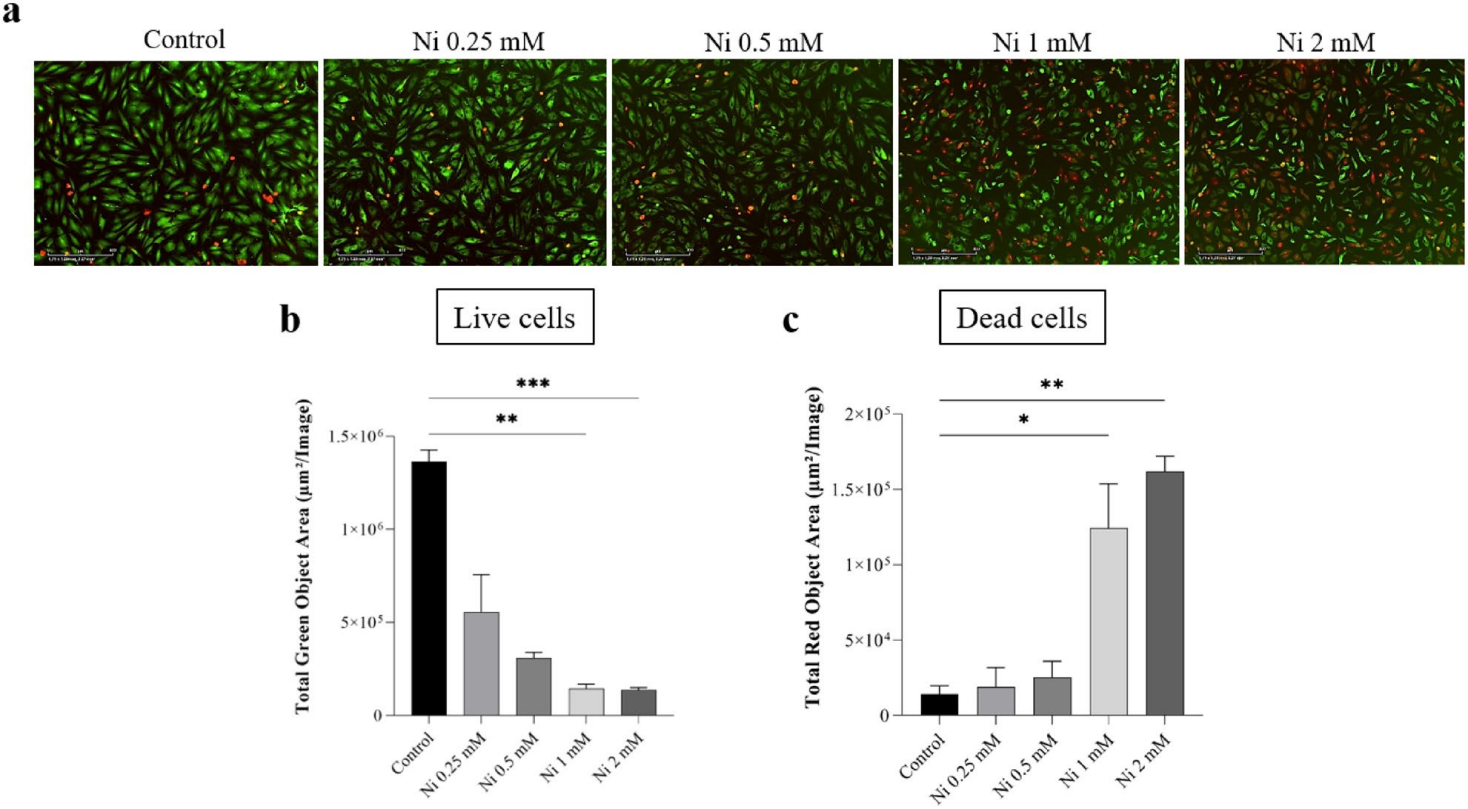
Analysis of Ni cytotoxicity after 24 h of cultivation with osteoblasts: (**a**) representative fluorescent images of green stained live cells and red-stained dead cells in presence of Ni (0.25 mM–2 mM) and in control sample (without Ni). Total green (**b**) and red (**c**) object area (µm^2^/Image) was calculated to determine cytotoxic effects on cells (*p < 0.05, **p < 0.01, ***p < 0.001).

As the first step for further analyses, Ni cytotoxicity tests were performed for OB-OA cells, to determine two concentrations on the borders of the effect: a lower one at which non-significant cytotoxic effects already occur, and a higher one—the first concentration with significant increase in dead cell count (Fig. 1).

After 24 h, in the presence of 0.25 mM, 0.5 mM, 1 mM and 2 mM Ni concentrations, changes in the number of live cells (green staining) and the number of dead cells (red staining) were analysed and expressed as total green/red object area (µm^2^/Image). The concentration dependent trend with the increased dead cell number was observed (Fig. 1a). A significant border change in the number of living cells occurred at a Ni concentration of 1 mM (p < 0.01) (Fig. 1b). In the primary patient osteoarthritic cells, there was an almost ninefold reduction in live cell signal at 1 mM concentration, compared to the control. As for the effects of 0.5 mM and 1 mM concentrations compared to the control, the death cells count increased from 1.8-fold at 0.5 mM to 8.6-fold in concentration of 1 mM Ni (Fig. 1c).

### Effects of different Ni concentrations on cell cycle and cell viability

To study the cytotoxicity of Ni^2+^ ions, the effects of several Ni concentrations on the cell cycle of OB-OA cells were evaluated. Cell cycle histograms show normal distribution of cell cycle phases in control and Ni-treated cells at all concentrations (see Supplementary Fig. S1 online). However, in cells treated with Ni at concentrations of 0.5 mM and higher, there is a clear increase in cells in the sub-G1 phase indicating apoptotic and dying cells (Table 1).

**Table 1:** Cell population percentages (%) in samples samples treated with 0 mM of Ni (control), 0.25 mM, 0.5 mM, 1 mM and 2 mM concentrations.

	Control	Ni 0.25 mM	Ni 0.5 mM	Ni 1 mM	Ni 2 mM
sub G1 (P9) (%)	9.06 ± 3.55	26.68 ± 11.35	26.62 ± 4.14	35.60 ± 5.45	47.65 ± 0.74
G1 (P7) (%)	75.36 ± 12.12	61.89 ± 3.67	63.70 ± 3.93	57.22 ± 5.58	32.32 ± 4.04
G2 (P8) (%)	6.39 ± 1.55	4.26 ± 1.41	3.64 ± 0.69	2.45 ± 0.51	1.43 ± 0.35

The values mentioned as mean ± SD.

**Figure 2:**
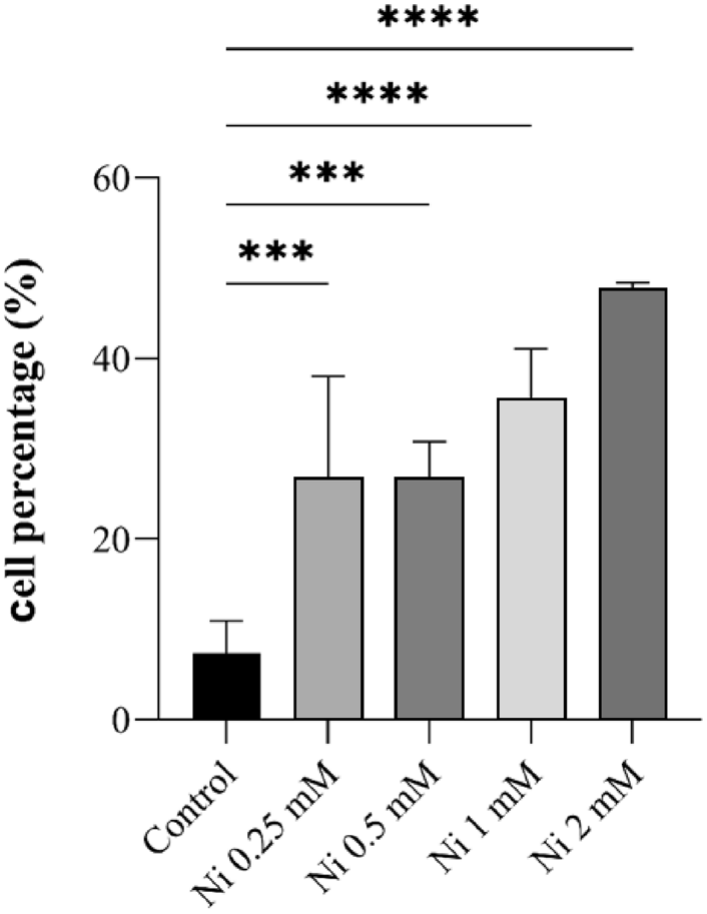
Percentage of dead cells in samples without (control) and with 0.25 mM, 0.5 mM, 1 mM and 2 mM of Ni concentrations. Statistical analysis was performed to reveal the differences in %cells between different Ni concentrations (***p < 0.001, ****p < 0.0001).

The reduced sizes of the total cell populations were also observed, which is related to the reduced cell viability confirmed in further experiments. Upon Ni treatment, a concentration-dependent increase in the number of apoptotic/dead cells represented by sub-G1 phase was observed. For each Ni concentration, a statistically significant change in number of sub-G1 cells was detected. Whereas only 7% of these cells were found in control sample, for Ni 0.25–2 mM the percentages ranged from 27 to 48% (Fig. 2).

The results of cell cycle showed that Ni concentrations up to 0.25 mM have little effect on cell viability, while concentrations of 0.5 and higher affect cell viability. Based on this fact, the next experiments are focused on the concentrations of 0.5 mM and 1 mM as the “borderline” concentrations.

### Cell proliferation analysis

For the selected concentrations of 0.5 mM and 1 mM of Ni, a proliferation analysis was performed. During the experiment, the morphological state of the cells was checked at certain time points of 24 h, 48 h and 72 h (see Supplementary Fig. S2 online). The occurrence of a larger number of floating cells visible in Ni-loaded samples (for 48 h and 72 h) could indicate that there was a reduction in the number of living cells. This fact was confirmed by the following resazurin test.

**Figure 3:**
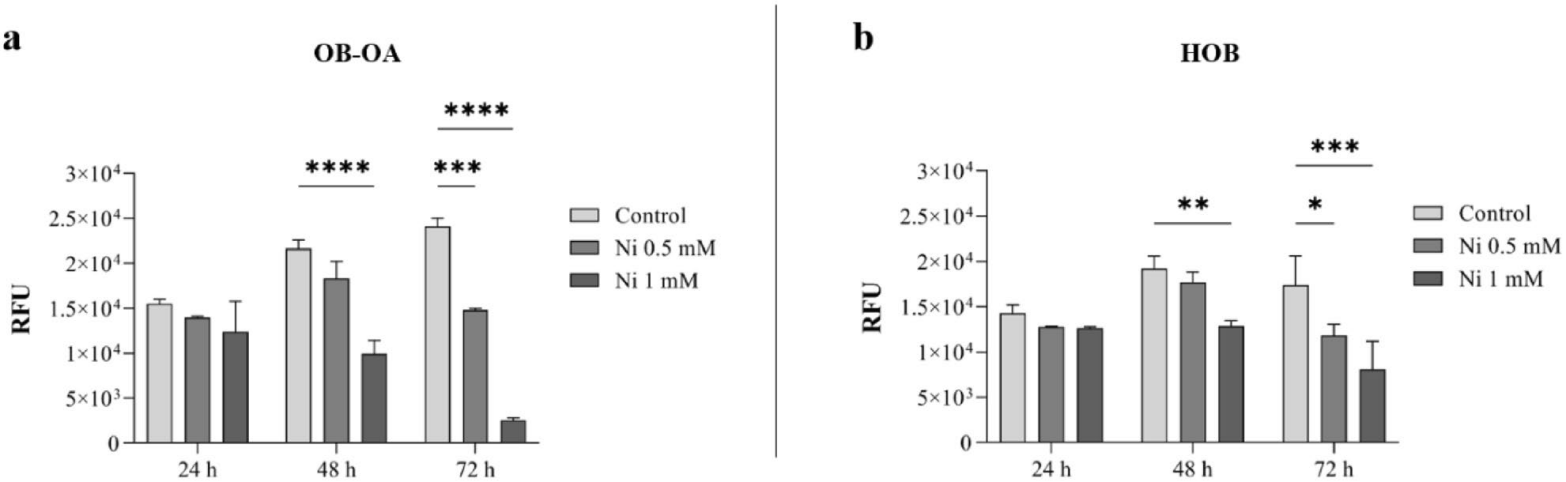
Measurement of cell proliferation with/without (control) Ni: (**a**) measurement of metabolic activity of OB-OA cultured with 0.5 mM and 1 mM of Ni for 24 h, 48 h and 72 h, (**b**) measurement of metabolic activity of HOB cultured with 0.5 mM and 1 mM of Ni for 24 h, 48 h and 72 h. Statistical analysis was performed with *p < 0.05, **p < 0.01, ***p < 0.001, ****p < 0.0001.

Metabolic activity of the cells was measured using the resazurin test performed at the same time points (24 h, 48 h and 72 h of cell cultivation with Ni) (Fig. 3). The analysis was performed for patient OB-OA, with the additional comparison with physiological HOB to observe possible differences in the sensitivity to Ni between different types of the cells.

Measurement of the metabolic activity of patient OB-OA cells showed that 1 mM concentration of Ni caused a highly statistically significant reduction in the cell metabolism already after 48 h (p < 0.0001), while after 72 h the effects were also observed at lower concentrations of 0.5 mM (p < 0.001) (Fig. 3a). Regarding physiological HOB cells, a significant decrease in the cell activity after 48 h for a Ni concentration of 1 mM (p < 0.01) was detected compared with the control sample (Fig. 3b). After 72h of cultivation, a reduction was observed at both concentrations of 0.5 mM and 1 mM, with the differences becoming more visible as the concentration increased.

Analysing the effect of Ni^2+^ ions on the proliferative ability of cells loaded with 0.5 mM of Ni over time, we found that a trend of the increase in the cell activity was still observed after 48 h, for both cell types. Measurement of the cell metabolic activity after 72 h revealed a gradual suppression of proliferation compared to 48 h values: a decrease by 33% and 19% for HOB and OB-OA, respectively. For the higher concentration of 1 mM, the proliferation of OB-OA cells was inhibited already at 48 h time point of cultivation with Ni, while the decreasing trend was maintained even after 72 h. Compared to the 24 h value, the cell activity decreased by 19.5% after 48 h and after 72 h—by another 60%. Interestingly, in the case of HOB cells, a reduction by 37% in proliferation was observed only 72 h after cultivation with Ni.

### Ni mapping in cells

#### Ni distribution in cell compartments

**Figure 4:**
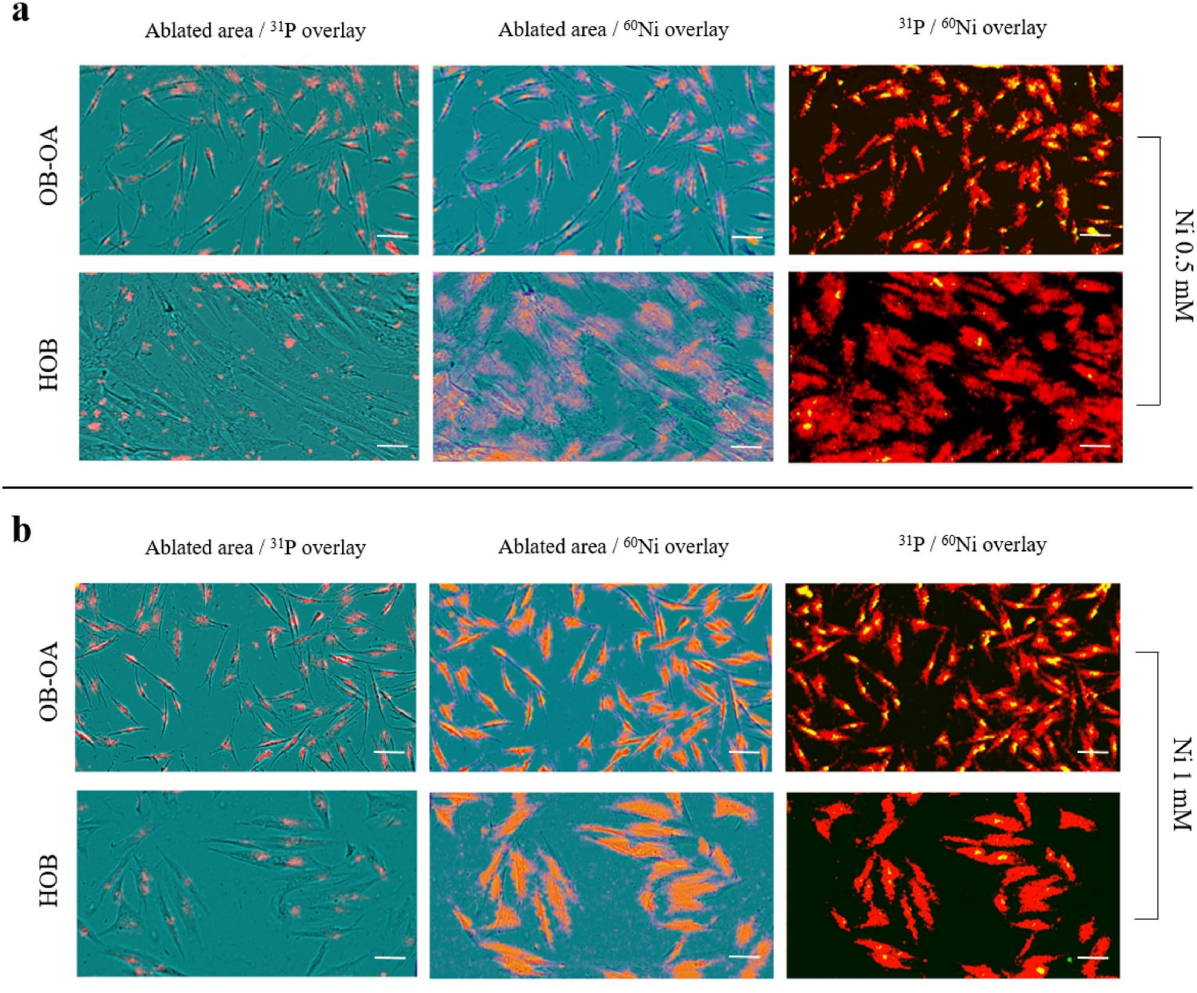
Distribution of 31P and 60Ni in HOB and OB-OA cells at Ni^2+ ^concentrations of 0.5 mM (**a**) and 1 mM (**b**). Overlays of ablated ^31^P and ^60^Ni with IncuCyte bright field image of cells are shown as ablated area/^31^P overlay and ablated area/^60^Ni overlay, respectively. On maps of ^31^P/^60^Ni overlay, the presence of ^31^P is shown in green, ^60^Ni in red, ^31^P + ^60^Ni overlapping in yellow. Scale bar = 100 µm.

Mapping of Ni distribution in cells was performed using LA-ICP-MS, with prior imaging of cells using IncuCyte^®^. For these samples, ^31^P and ^60^Ni isotopes were always ablated and their distribution is presented in the form of 2D maps (see Supplementary Fig. S3 online). Thanks to prior cell imaging, it was possible to find the exact position of the ablated area and to overlap the ablation map with the image of the cells. Moreover, the position of each isotope in an individual cell was found, and therefore the distribution maps of the elements were superimposed on each other (^31^P/^60^Ni overlay). Assuming that the position of ^31^P could indicate the location of the nucleus in the cell (marked in green). The distribution of ^60^Ni was imaged in red. In case of ^31^P and ^60^Ni overlapping, a yellow-coloured area was observed. Analysis of ^31^P and ^60^Ni isotopes was performed in both cell types—HOB and OB-OA, while two concentrations of Ni—0.5 mM and 1 mM—were tested during the first experiment (Fig. 4).

**Figure 5:**
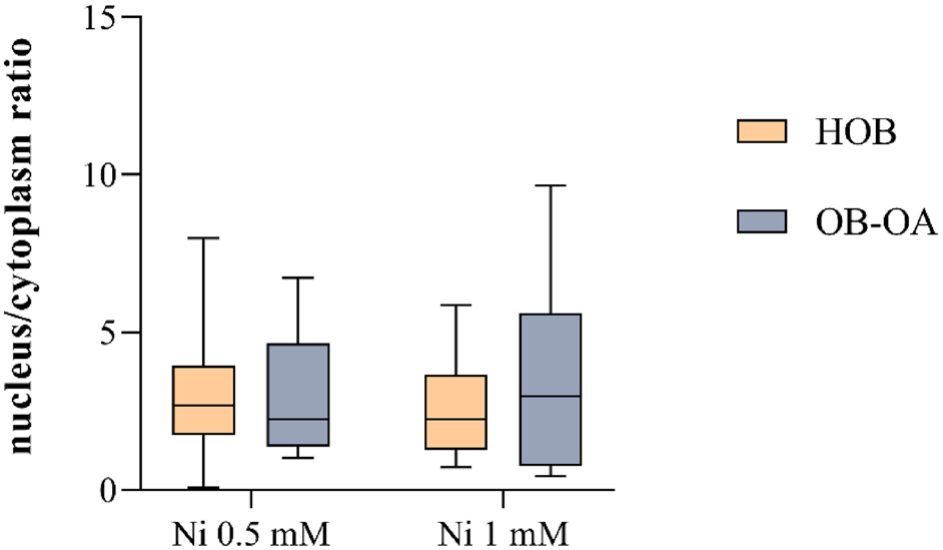
Distribution of Ni in HOB and OB-OA loaded with 0.5 mM and 1 mM concentrations measured after 24 h of cultivation with Ni^2+^ . Results are expressed as nucleus/cytoplasm ratio calculated from 60Ni intensities.

For both cell types, Ni was shown to be distributed throughout the cell, i.e. in the nucleus and cytoplasm. In ^31^P/^60^Ni maps, it was generally noted that ^31^P signal in HOB samples was overlayed by ^60^Ni maps so no clear signal was observed for ^31^P + ^60^Ni. Thanks to this, locating the exact area of the nuclear (using ^31^P) and cytoplasmic (^60^Ni with subtraction of the ^31^P area) compartments was possible. The amount of Ni in individual cell compartments was calculated as intensity values (cps). The results were expressed as the nucleus/cytoplasm ^60^Ni intensity ratio (Fig. 5).

Analysis of the nucleus/cytoplasm ratio within individual concentrations revealed that there is no significant difference between the distribution of Ni in HOB and OB-OA at 0.5 mM and 1 mM. At a lower concentration the ratio value for HOB is slightly higher than for OB-OA (median 2.68 and 2.24 for HOB and OB-OA, respectively), while at 1 mM the result is the opposite (2.24 and 2.97).

#### Ni absorption by osteoblasts in time

**Figure 6:**
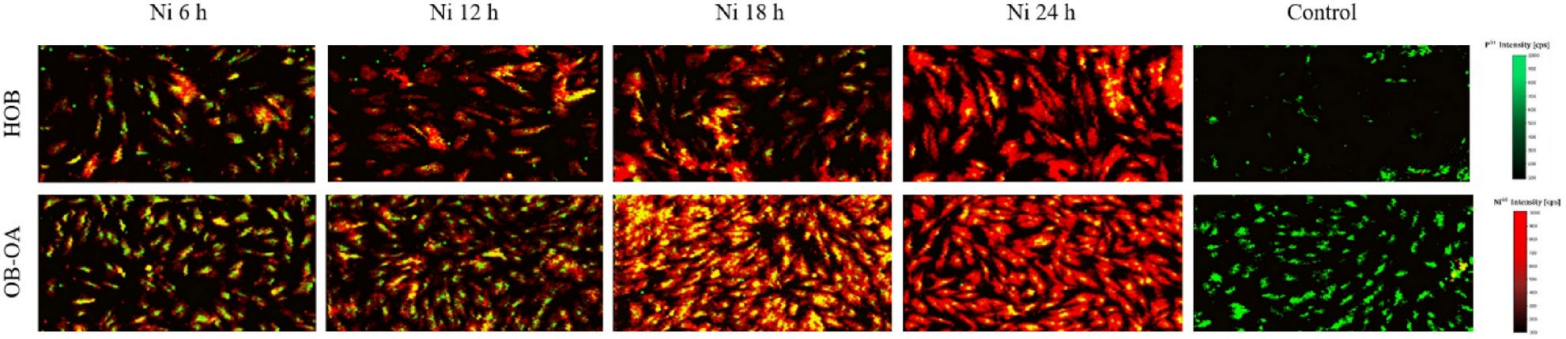
^31^P (green) and ^60^Ni (red) distribution in HOB and OB-OA after 6 h, 12 h, 18 h and 24 h of cultivation with Ni. ^31^P and ^60^Ni overlapping is presented in yellow. Control sample (cells without Ni) was added to prove the absence of Ni.

After proving of Ni^2+^ ions penetration and accumulation in the cells, a monitoring of the absorption of Ni^2+^ ions over time was crucial. In particular, the main aim was to trace Ni in the cell in time and monitor cell saturation with Ni. For a better visualisation of the distribution, a higher concentration of 1 mM was used, and LA-ICP-MS was performed on cells after 6 h, 12 h, 18 h and 24 h of cultivation with Ni (Fig. 6). Control samples (cells without added Ni) showed the absence of Ni on the ablation maps.

From the obtained results, it can be observed that after 6 h there was a partial accumulation of Ni in the cells, both in the nucleus (presence of yellow-marked ^31^P + ^60^Ni overlapping), and also in the cytoplasm (red-coloured ^60^Ni cells). However, a prevalence of cells with only ^31^P labelling in nuclei (green) can still be seen. Over time, there was an increase in intensities for ^60^Ni in all cellular compartments. The biggest difference was observed between 12 and 18 h, when Ni^2+^ ions were already present in all cell nuclei after 18 h of cultivation. Likewise, there was an increase in the “red-labelled area”—a sign of the saturation of the cytoplasmic compartment with nickel. This may be caused by high concentration of Ni ions throughout the cell, with the result of ^31^P signal “masking”.

**Figure 7:**
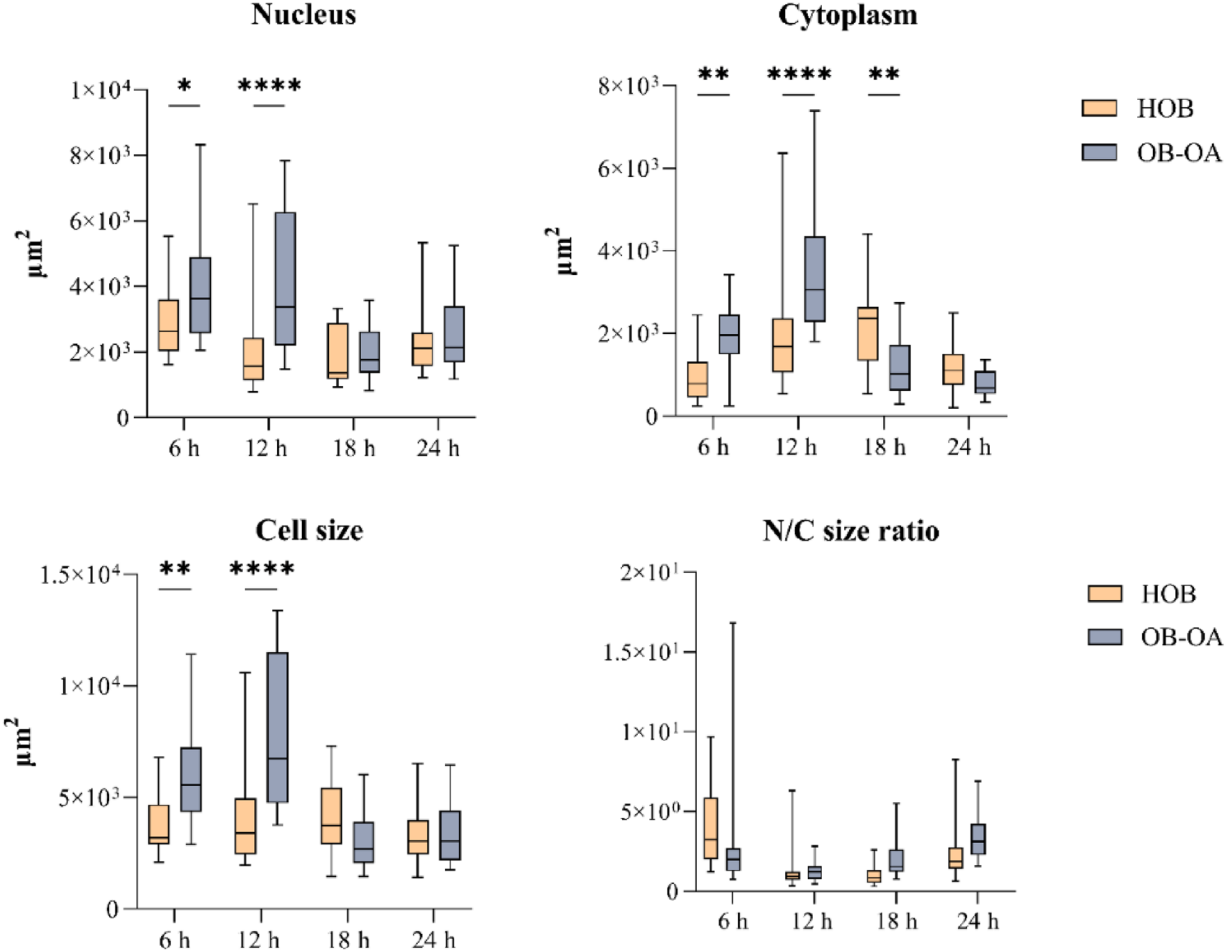
Analysis of HOB and OB-OA area of the cell, nucleus and cytoplasm and nucleus/cytoplasm (N/C) size ratio calculated from the ablation maps. Statistical analysis of the difference between two cell lines were performed in the time points of 6 h, 12 h, 18 h and 24 h.

Distribution maps were also used to check the size of the cells, the nucleus and cytoplasm, and to monitor their changes over time (Fig. 7). As for the size of the cells, in case of HOB there were no visible changes over time (the median is in the range of 3.0–3.7 × 10^3^ µm^2^). For OB-OA, an increase in cell area was detected after 12 h compared to 6 h—the values were 5.6 × 10^3^ µm^2^ for 6 h and 6.8 × 10^3^ µm^2^ for 12 h. However, over time these values were reduced to 2.7 × 10^3^ µm^2^ (for 18 h) and 3 × 10^3^ µm^2^ (for 24 h). From these results it can be seen, that significant differences in size between HOB and OB-OA (cell size, nucleus and also cytoplasm) were visible only at the first two time points.

At the same time points, significant differences were also observed in the case of nuclei size, with OB-OA nuclei generally having larger areas than HOB – 3.6 × 10^3^ µm^2^ vs. 2.6 × 10^3^ µm^2^ for 6 h (p < 0.05) and 3.4 × 10^3^ µm^2^ vs. 1.6 × 10^3^ µm^2^ for 12 h (p < 0.0001), respectively. Interestingly, in the following times, there was an equalization of the sizes of the nuclei of both cell types, where at the time of 24 h they both had an equal median value of 2.1 × 10^3^ µm^2^.

Changes in cytoplasmic size are consistent with the parameters described above, which support the fact that the change occurs in all cellular compartments. For OB-OA cell type, a period between 12 and 18 h could be defined as the breakpoint, when a trend of increasing cytoplasmic size (time points 6–12 h) was followed by a decreasing trend (18–24 h). These changes were also reflected in the values of nucleus/cytoplasm (N/C) size ratio, when the turning point between 12 h and 18 h also changed the trend, but in the opposite direction. Comparison of cytoplasmic sizes of two cell lines showed significant differences for 6 h, 12 h and 18 h, with the crucial difference observed at 12 h time point (p < 0.0001). Interestingly, a turning point for HOB was “delayed” and set at a time period between 18 and 24 h. By analysing these results, it can be assumed that OB-OA could be able to respond to the presence of Ni more rapidly with more effective adaptation to changing conditions than HOB.

**Figure 8:**
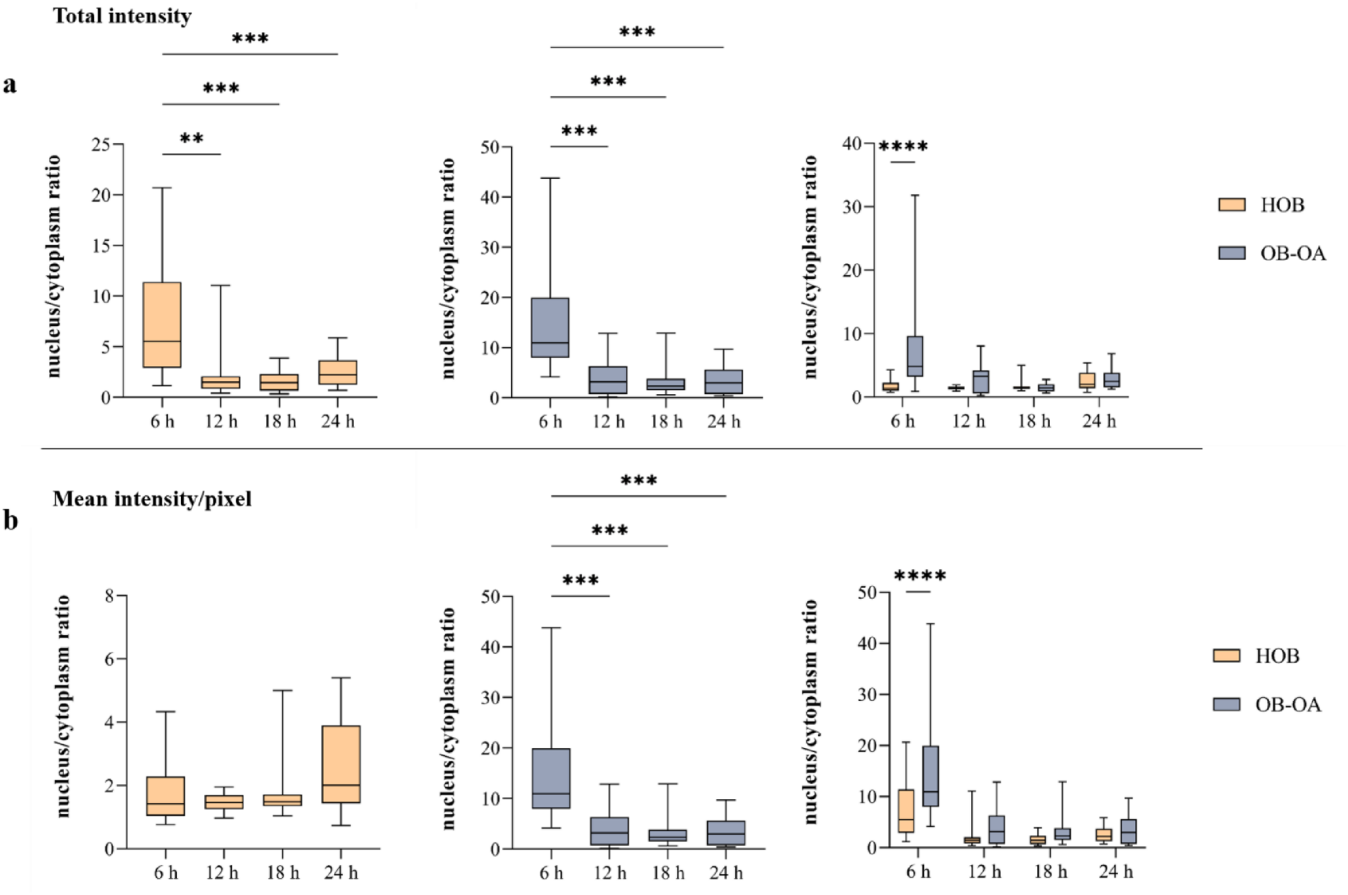
Ni distribution in cell expressed as nucleus/cytoplasm ratio in HOB, OB-OA cells, and the comparison of Ni distribution between HOB and OB-OA cell lines: (**a**) total cell intensity, (**b**) mean intensity/pixel. Statistical analysis was performed to examine if there are differences between different time points and cell lines (*p < 0.05, **p < 0.01, ***p < 0.001, ****p < 0.0001).

In order to understand in which part (nucleus vs. cytoplasm) nickel accumulates at different time points, Ni intensities were calculated from the original isotope matrices. The results were expressed as the ratio of nickel intensity in the nucleus/cytoplasm (Fig. 8). Figure 8a represents the intensity ratio results, which were calculated as a sum of intensities of all pixels corresponding to a particular cell part. Furthermore, because of the differences in area sizes of HOB and OB-OA observed during the previous experiment, Ni intensities were also referred to nucleus and cytoplasm size and expressed as mean intensity/pixel to avoid size effects (Fig. 8b).

Analysing total intensity results, there were significant changes in the nucleus/cytoplasm ratio over time for both cell lines. For 6 h, the value was the highest in both cases (5.54 and 10.92 for HOB and OB-OA, respectively), reflecting a larger amount of Ni found in the nucleus. For time 6–18 h, the value of the ratio gradually decreased, i.e. the amount of nickel in both cell compartments gradually equalized, with the mean values of 1.50 and 3.18 after 12 h and 1.47 and 2.36 after 18 h for HOB and OB-OA, respectively. It was also observed that after 24 h the trend was changed, and followed by an increase in the ratio values to 2.24 and 2.97 for HOB and OB-OA. Within individual cell lines, a significant difference in the nucleus/cytoplasm ratio was found for both OB-OA and HOB, with the statistically highest value measured at 6 h. Comparison of Ni distribution between the two cell lines revealed that a significant difference was only observed at the 6 h time point, where the ratio of Ni in the nucleus was higher in OB-OA than in HOB (p < 0.0001), which may by connected with the highest value for nucleus size.

The results converted to mean intensity/pixel values had comparable trends in the case of OB-OA cells. However, the largest differences were observed in the case of HOB, where no differences in N/C ratio were detected between individual time points. However, this finding may be influenced by the fact that the changes in cell size induced by the presence of Ni were not as significant as in the case of OB-OA (Fig. 7).

## Discussion

### General cytotoxicity of Ni: proliferation and cell cycle changes

The release of metal ions from implanted materials is a topical issue for contemporary implantologists. Nickel, as an element with strong immunomodulatory and, in general, cytotoxic effects, is particularly in the centre of interest. Thanks to the interdisciplinary connection of implantology, immunology and toxicology, it is among the most studied metals from the point of view of both *in* *vitro* and *in vivo* approaches.

In present work, we focused on studying the local effects of Ni—its impact on primary patient cells from osteoarthritic patients. From our point of view, this cellular model appears to be one the most appropriate ways to simulate real conditions; these cells will actually come into contact with metal after joint replacement surgery. To reveal the cytotoxic effects of Ni, two methods of cell viability measurement were used—live/dead cell imaging using fluorescent dye with the following confirmation of this method using the more conventional flow cytometry method. The results of both techniques showed similar trend regarding Ni cytotoxicity—low level of cytotoxicity was caused by concentrations of 0.25 mM and 0.5 mM, whereas crucial changes (rapid increase in dead, probably apoptotic, cell number) were observed between concentrations of 0.5 mM and 1 mM. By analyzing the distribution of cells across different phases, Sub G1 population typically represents cells that have undergone DNA fragmentation, which often occurs during apoptosis (programmed cell death). The G2 phase refers to cells with doubled DNA content compared to G1, and is a critical stage where cells prepare for division. Cell cycle analysis did not reveal the arrest in any phase, but increasing sub-G1 population showing apoptotic and dead cells and decreasing total number of cells, with the decrease in G2 phase. These results indicate significant reduction of cell proliferation, especially in Ni concentrations of 0.5 and 1 mM. From the above-mentioned results, we can state that Ni concentrations higher than 0.5 mM bring significant changes in human osteoarthritic cell behaviour.

Earlier works dealt with the study of cytotoxic effects on osteoblast-like tumor cells and murine cell lines. Hallab et al. proved that cytotoxic effects of Ni^2+^ on MG-63 osteoblastic cell line occurred at a concentration higher than 0.5 mM. In their study, a decrease of cell viability and cell proliferation, as well as cytoskeletal morphology reorganisation, inhibition of procollagen α1 expression and significant changes in pro-inflammatory IL-6 secretion was noted^12^. Another study by Kanaji et al. reported that Ni^2+^ ions had significant cytotoxic effect on MLO-Y4 osteocytes at concentration higher 0.1 mM^7^. The authors also dealt with the analysis of cell death using Enzyme-Linked Immunosorbent Assay (ELISA), which showed that apoptosis was the predominant type of cell death in case of nickel toxicity.

The effects of Ni on the cell cycle have been described in other studies on bone cells, where similar effects were observed at higher Ni concentrations. D’Antò et al. studied *in vitro* effects of higher concentrations of Ni^2+^ ions (up to 5 mM) on human osteosarcoma cells^13^. Authors stated that Ni induced a dose and time dependent cytotoxicity and inhibition of cell proliferation. Moreover, significant changes in cell cycle distribution in both cell lines were reported after 24 h exposure to 2 mM of Ni^2+^. An earlier study by Sun et al. described the effects of Ni on reactive oxygen species (ROS) during 17/2.8 osteoblast-like cell metabolism and differentiation^8^. The results after 3 days of cultivation showed that significant cytotoxic effects appeared at a concentration of 0.3 mM, while the TC_50_ value was 0.6 mM.

The effects of Ni can vary depending not only on the concentration, but also on the type of cells used in the study. In general, tumour cells are characterised by an alternative way and the rate of metabolism, extended beyond the Warburg effect—despite the presence of oxygen and fully functioning mitochondria, the rate of glucose uptake dramatically increases with accumulation of lactate^14–16^. For this fact, it may be the reason that tumour cells are more resistant to the presence of lower concentrations of Ni. According to our best search results, this is the first study evaluating the toxic effects of Ni^2+^ ions on osteoarthritic patients’ cells, which represent the most suitable environment for studying the cytotoxicity of implant-released ions.

Proliferation analysis provided another interesting results regarding differences in response to Ni between patient osteoarthritic and commercially available physiological osteoblastic cell lines. From the obtained results we can conclude, that OB-OAs seem to be more sensitive to higher concentrations of Ni (both 0.5 mM and 1 mM) than HOBs. A study by Válková et al. describes the effect of a Ni-containing material (nitinol) on physiological and osteoarthritic osteoblasts^17^. The results show that osteoarthritic osteoblasts had a higher expression of inflammatory interleukin-1β (IL-1β) and IL-8 after contact with the material than physiological osteoblasts. More recent study by Štefančík et al. also reports the differences in the reaction of physiological and osteoarthritic osteoblasts to Ni nanoparticles, which were manifested both by changes in cell sizes and actin and tubulin expression^18^. Our study shows that the decisive role in Ni cytotoxicity is played by Ni ions, which are released from any Ni-containing material^19^, no matter what form it is (the ions themselves, nanoparticles, or metal material). We have also proved that there is indeed a higher grade of sensitivity of osteoarthritic osteoblasts to Ni which is consistent with previous studies.

The differences in OB-OA and HOB response could be explained by the fact that OB-OA cells were already exposed to inflammatory conditions in the body of osteoarthritic patient, which apparently can affect the behaviour of the cells in vitro. Although osteoarthritis has long been considered a cartilage-driven non-inflammatory type of arthritis, it has now been confirmed that it represents a much more complex disease with a great role of inflammatory mediators released by synovium, cartilage, and bone^20–25^. Proinflammatory cytokines, including tumour necrosis factor-α (TNF-α), interleukin-1 (IL-1), IL-6, interleukin-17 (IL-17), interleukin-21 (IL-21), are capable of affecting the behaviour of bone cells leading to suppression of osteoblast activity and function^25–27^.

Another possible explanation is the fact that every patient comes into direct contact with Ni during his life, whether contained in air, food or water^1,2,28^. Accumulation of Ni in organs was confirmed, e.g. in the brain, stomach, liver, kidneys, lungs and heart^29,30^ with oxidative stress, mitochondrial dysfunction and epigenetic changes being described^28^. Even thoug, literature expects the whole body accumulation in the tissues, the concentration of Ni in bones was not hence discribed^31^.

### Ni mapping using LA-ICP-MS

LA-ICP-MS is a powerful tool for determination of the spatial distribution of elements in various solid samples. This method is very useful in the analysis of biological samples in which imaging of metals, metalloids, isotopes and biologically important elements helps to explain a biological and biomedical processes^32^. The first experiments on cell analysis by LA-ICP-MS were performed about 10 years ago. Giesen et al. used an iodine dye for fibroblast cell imaging. The dye immediately after staining reaches predominantly the nucleus of the cells. Based on the distribution of iodine, it was possible to distinguish the nucleus from the cytoplasm^33^. Other publications devoted to the distribution of elements in cells have been focused on imaging of the distribution of silver and gold nanoparticles in individual fibroblast cells^34^ or on monitoring Gd-based contrast agents in the field of cellular immunotherapy^35^. Nowadays, cell analysis by LA-ICP-MS is finding increasing applications due to better resolution and sensitivity (Van Acker et al. 2019).

In this study, we used LA-ICP-MS method for monitoring of time-dependent Ni accumulation in the cell with the focus on distinguishing the changes in concentration of the Ni in nucleus and cytoplasm. for analysis of Ni distribution in bone cells, both patient osteoarthritic and physiological osteoblasts. According to our best search results, there is no work dealing with the analysis of the Ni content in the cell in time intervals so far. It is also the first study that describes the movement of Ni between cellular compartments in time, and thus represents another possibility of using the conventional LA-ICP-MS method in biological applications. Moreover, using this method the changes in cell size were possible to determine, as well as changes in the size of the nucleus and cytoplasm affected by the presence of Ni.

**Figure 9:**
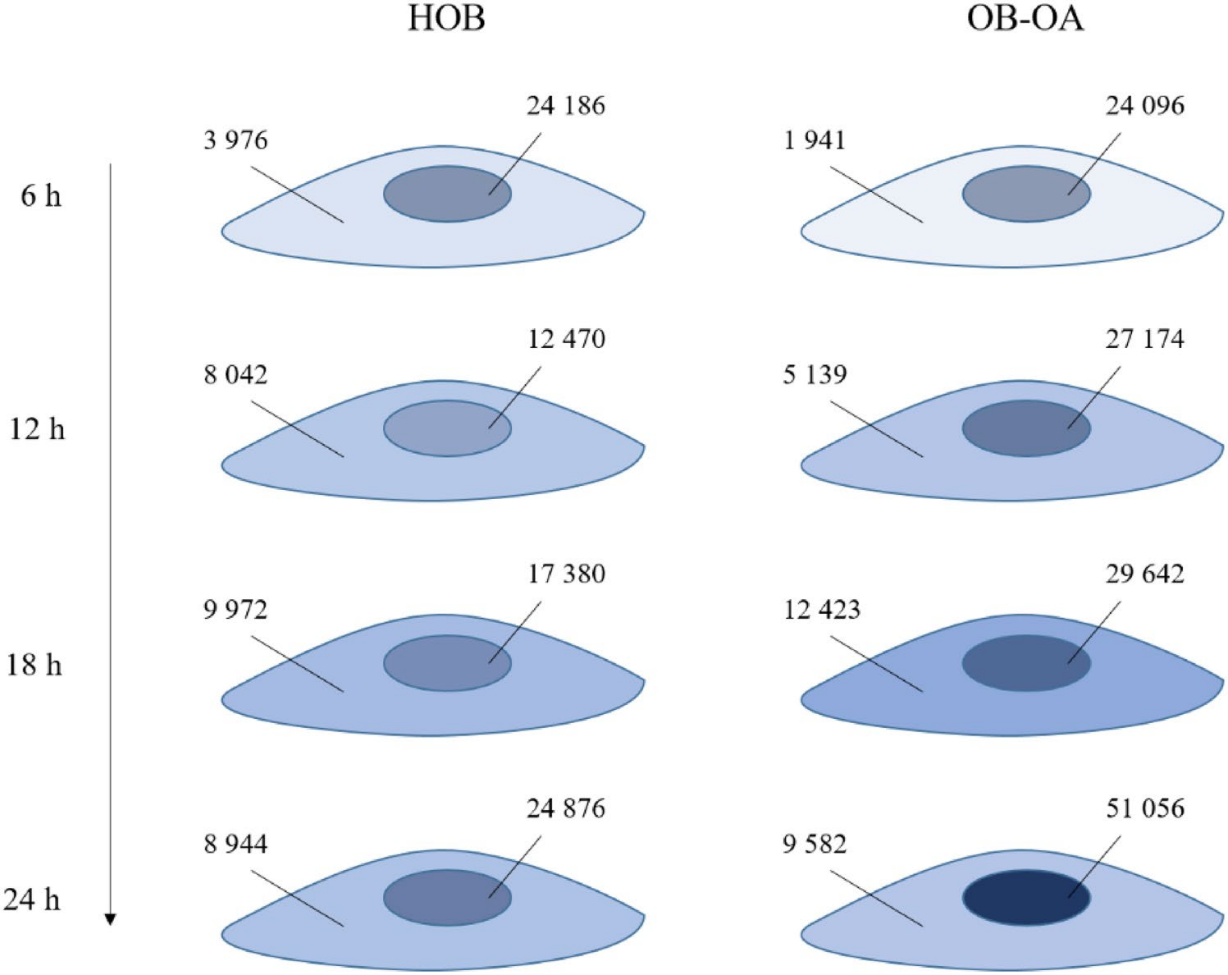
Schematic representation of ^60^Ni summary intensities (in cps) in the cell compartments (nucleus and cytoplasm) for HOB and OB-OA in time (6 h, 12 h, 18 h and 24 h). Intensity of a blue colouring also reflects Ni concentration in nucleus and cytoplasm.

From the achieved results, we can state that in OB-OA more rapid changes in the size of both the nucleus and cytoplasm (thus the cell size) were observed over time. This finding may indicate that OB-OA cell type is capable of faster adaptation to changing conditions than physiological HOB. Analysis of Ni content in time showed changes in nucleus/cytoplasm ratio of Ni concentration, which may indicate that a continual exchange of Ni between nucleus and cytoplasm is taking place. To verify this hypothesis, the average Ni intensities in the nucleus and cytoplasm were calculated for each time point and both types of cells, and expressed graphically for greater clarity (Fig. 9).

From the schematic representation, it can be observed that the largest increase in total Ni amount occurred in the time period of 0–6 h. Therefore, it can be assumed that exposure to Ni (within 6 h from the beginning) leads to the largest accumulation of Ni^2+^, both in OB-OA and HOB. Our theory is that it could be caused by rapid changes of the conditions that the cells had to adapt to, which ensured the rapid uptake of Ni into the cells, primarily into the nucleus. The absorption of Ni by cells is a remarkable process; the absorption speed is not constant throughout the time, where so-called fast and slow phases can be distinguished. Refsvik and Andreassen reported that, for example, in human epithelial kidney cells, the fastest uptake of Ni by cells occurs during the first 2 h of culture, while a slow phase of the uptake lasts for several hours follows^36^. Additionally, Edwards et al. noted that the rate of Ni uptake varies among different cell types^37^. Author states that macrophages, compared to fibroblasts, have a lower rate of nickel absorption, which is probably affected by both cell size and the presence of a different Ni uptake mechanisms^37,38^. In our case, we can state that the fast phase of Ni uptake by osteoblasts takes place during the first 6 h after Ni exposure into the cells.

Furthermore, from the changing intensity values for Ni in the nucleus and cytoplasm, it can proved that there is an active transfer of Ni^2+^ ions between these cell parts (e.g. 6 h and 12 h for HOBs). Moreover, comparing the total amount of Ni at these time points, it can be observed that there is also an extracellular transport of Ni (the total amounts for the nucleus and cytoplasm are 28,162 cps and 20,512 cps for 6 h and 12 h, respectively). The fact of Ni excretion by cells into the environment has been noted in other studies. Assays were performed as follows: after culturing with Ni or Ni-containing materials, the cells were transferred to Ni-free culture medium, where the Ni concentration in the new medium was then measured. Ke et al. monitored Ni transport in A549 cells by fluorescent labelling with Newport Green dye mixture^39^. Excretion of Ni by cells was recorded after 10 h of transfer to pure medium, with only trace concentration of Ni in cells detectable after 16 h; after 24 h Ni had already completely disappeared from the cell. Veverkova et al. studied the uptake and excretion of Ni by human umbilical vein endothelial cell (HUVEC) after culturing on NiTi plates, with regard to the material surface treatment^40^. The author states that after removing the material from the cells and replacing the culture medium with a pure one, values of 11.4 μg/l and 2.6 μg/l of nickel in the clean medium were measured for H_2_-coated and He-coated NiTi. Our study differs from the above and describes the back-excretion of Ni even in the constant presence of Ni in the culture medium.

After 24 h, a noticeable difference in Ni amount between two cell types can be observed—its amount in OB-OA was 2 × higher than in HOB, i.e. OB-OA are capable of larger Ni accumulation than physiological osteoblasts. While the mechanism of nickel ion transport has been described in prokaryotes and fungi^41–44^, regulation of Ni uptake in mammalian cells has not yet been explained in detail^45^. In connection with the aforementioned factors potentially affecting the differences between OB-OA and HOB (inflammatory conditions in the patient’s body and previous exposure to nickel), there is a suggestion that these factors could also cause changes in some of the components of the OB-OA’s transport system, leading to a different ability to accumulate Ni compared to physiological osteoblasts. Since there is no publication dealing with the study of mechanisms of Ni transport and the differences between these cell lines, it can only be assumed that in OB-OA there may have been changes in the expression of either ion channels or ion transporters, causing hyperaccumulation of Ni in the nucleus. Nevertheless, a study by Valkova et al. reports much more significant changes in the pro-inflammatory expression profile and extracellular matrix components of osteoarthritic osteoblasts in contact with Ni-containing material than in physiological osteoblasts^17^, which correlates with our hypothesis of differences in the expression of various proteins between the studied cell lines. From the above mentioned results, we can also presume that the larger accumulation of Ni in the OB-OA’s nucleus may be the reason for the higher sensitivity of OB-OA cells to Ni compared to HOB, which is manifested by a reduced proliferative ability (our results) and altered cell behavior described in comparable studies.

### Limitations of the study

We are aware of the limitations of the study, especially regarding the Ni concentration range relevant for the clinical use. The concentration of nickel Ni released from implants can vary widely depending on factors such as the type of implant, its composition, surface characteristics, and the surrounding biological environment. Actual release rates can vary over time and between individual patients, with the maximum in first 24 h due to mechanical stress of the surface. For instance, research by Hallab et al. examined the release of metal ions, including nickel, from orthopedic implants within the first 24 h^46^. They found that the initial release of nickel ions from implants can occur rapidly. In the study of Wataha et al. the nickel concentrations from the nickel-containing alloy (Ni–Cr) reached 48 μg/g near the implant, falling exponentially, dropped to less than 10 μg/g at 1.5 mm, to undetectable levels at 3–4 mm from the implants^47^. Regarding the continual load, a half of the amount released near to the implant was detected in less than 48 h. Its known that 10–50 μg/ml of nickel ions were necessary to cause total suppression of mitochondrial function in vitro experiments using fibroblasts, endothelial cells, and monocytes, which is in consensus to our findings on apoptotic death. Furthermore, in long-term use the increased concentrations of Ni may occur in case of implant corrosion and/or material defects, which can vary according to the nature and extent of the defect and the duration of implantation, with relevant material loss^48,49^. According to our best knowledge, the data about the material loss for Ni alloys does not exist but in case of titanium implants the modular neck hip implant revealed material loss of between 280 and 1640 µg after 5.5 × 10^6^ loading cycles—approximately 15 years^50^. Dong et al. measured the release of nickel from Nitinol due simulation of fretting corrosion in the Ringer’s solution, where the concantrations about 65 μg/L after 50,000 cycles were reported^51^. The research by Urban et al. examined the influence of surface defects on the release of nickel from orthopedic implants^52^. They found that surface irregularities and defects can enhance the release of nickel ions, potentially leading to increased exposure and adverse reactions in patients. This effect was proved in case of macroscopic corrosion of the alloy, where nickel was found well outside the limits of the normal range, with maxima of 300 µg/l in urine^53^. All above mentioned refers to different load due to direct contact with alloy or distant effect by ion release from the material, which will define behaviour of the cells.

## Conclusion

According to our best search results, this is the first study evaluating the toxic effects of Ni^2+^ ions on osteoarthritic patients’ cells, which are cells that come into direct contact with the metal after the application of a joint replacement and thereby represent the environment the most suitable for studying the cytotoxicity of implant-released ions. Using several methods, we demonstrated the low toxicity of Ni up to a concentration of 0.25 mM. We also demonstrated the dynamics and localization of Ni in cells and drew attention to the possible causes of toxicity—accumulation in the cells, localisation to the nucleus (DNA damage), the ability of the cells to release Ni over time, showing another possibility of using the conventional LA-ICP-MS method in biological applications. Moreover, using this method we were able to alternatively evaluate changes in cell size, as well as changes in the size of the nucleus and cytoplasm affected by the presence of Ni. The results of this study bring further knowledge regarding the response of bone cells to Ni as an element widely used in orthopaedic applications.

### Supplementary Information

Supplementary Information.

## Data Availability

The datasets used and/or analysed during the current study are available from the corresponding author on reasonable request.

## Abbreviations

DMEM/F-12: Dulbecco’s Modified Eagle’s medium/Ham’s nutrient mixture F12
ELISA: Enzyme-Linked Immunosorbent Assay
FBS: Foetal bovine serum
HOB: Human osteoblasts (physiological osteoblasts)
HUVEC: Human umbilical vein endothelial cell
IL-1: Interleukin-1
IL-1β: Interleukin-1β
IL-17: Interleukin-17
IL-21: Interleukin-21
IL-6: Interleukin-6
IL-8: Interleukin-8
LA-ICP-MS: Laser ablation inductively coupled plasma mass spectrometry
Ni: Nickel
OB-OA: Osteoblasts-osteoarthritis (patient osteoblasts)
OPG: Osteoprotegerin
PBS: Phosphate buffer saline
RANKL: Receptor activator of NF-κB ligand
TNF-α: Tumour necrosis factor-α
