# Supplementary material for: Cytotoxic effects and comparative analysis of Ni ion uptake by osteoarthritic and physiological osteoblasts
Source: Sci Rep. 2024 Jul 12;14:16133. doi: 10.1038/s41598-024-67157-9 (PMC11245524; doi:10.1038/s41598-024-67157-9)
Supplement: Supplementary file 1 — Supplementary Information. [file 41598_2024_67157_MOESM1_ESM.docx]

**Supplementary**


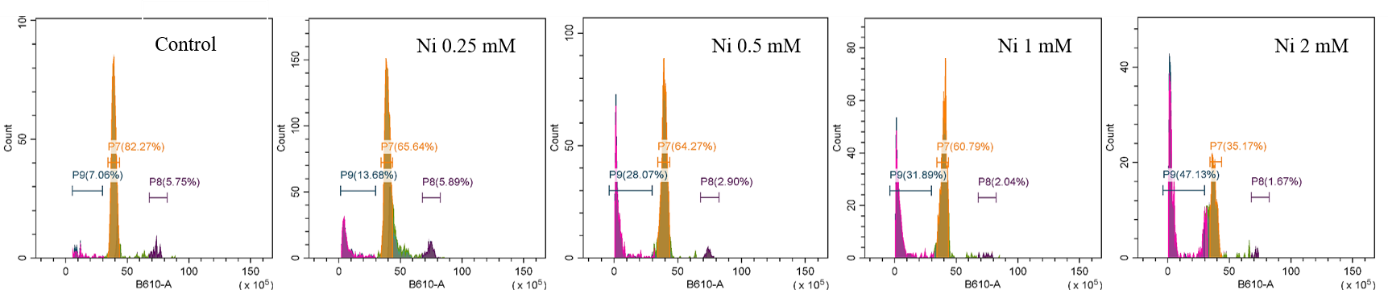
**Supplementary file 1:** representative flow cytometry plots of cell subpopulations: apoptotic (P9), G1 (P7) and G2 (P8) in samples treated with 0 mM of Ni (control), 0.25 mM, 0.5 mM, 1 mM and 2 mM concentrations. Sub-G1 represents apoptotic and necrotic cells.

**S1**:representative flow cytometry plots of cell subpopulations: apoptotic (P9), G1 (P7) and G2 (P8) in samples treated with 0 mM of Ni (control), 0.25 mM, 0.5 mM, 1 mM and 2 mM concentrations. Sub-G1 represents apoptotic and necrotic cells.

**Supplementary file 2:** morphology analysis of cells with/without (control) Ni ions. **a)** representative images of OB-OA cells cultured with 0.5 mM and 1 mM of Ni for 24 h, 48 h and 72 h. **b)** representative images
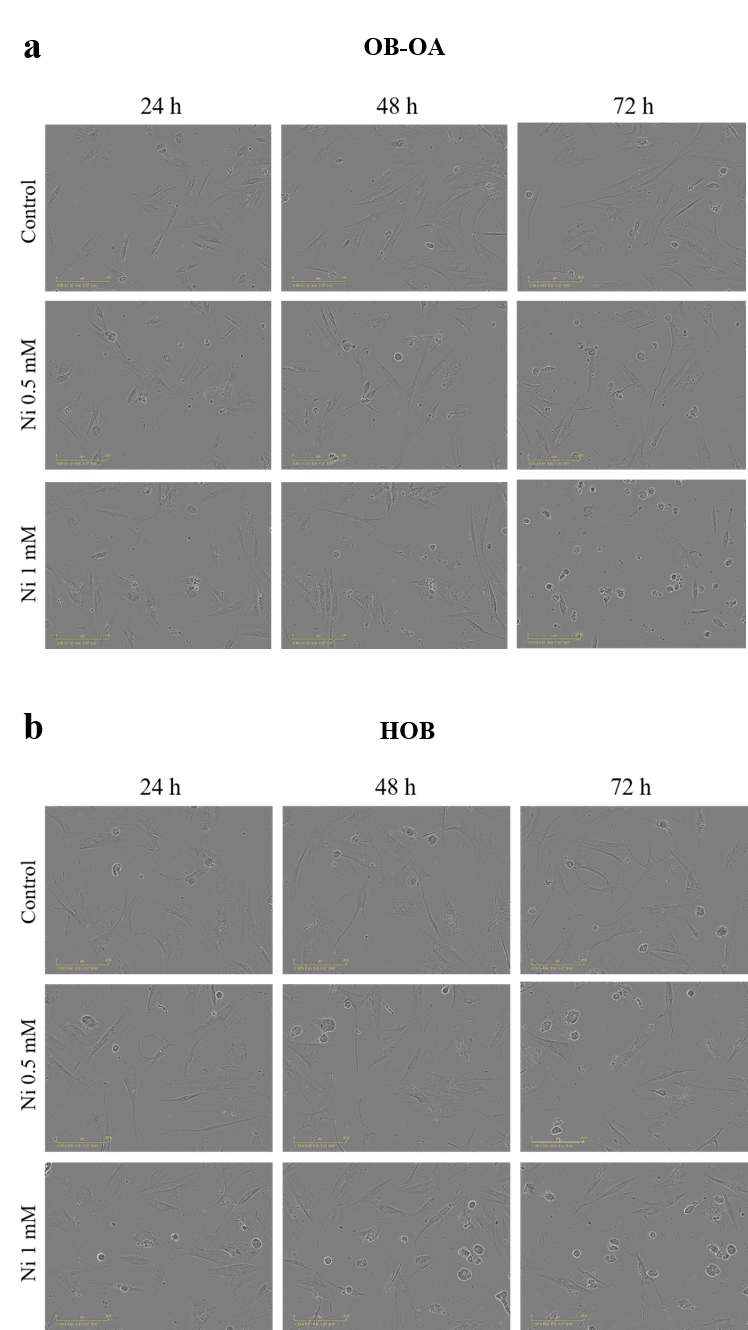
of HOB cells cultured with 0.5 mM and 1 mM of Ni for 24 h, 48 h and 72 h.

**S2**: morphology analysis of cells with/without (control) Ni ions. a) representative images of OB-OA cells cultured with 0.5 mM and 1 mM of Ni for 24 h, 48 h and 72 h. b) representative images of HOB cells cultured with 0.5 mM and 1 mM of Ni for 24 h, 48 h and 72 h.

**Supplementary file 3:** distribution of  ^31^P and ^60^Ni in HOB (a) and OB-OA cells (b) in control samples.

S3: distribution of ^31^P and ^60^Ni in HOB (a) and OB-OA cells (b) in control samples.


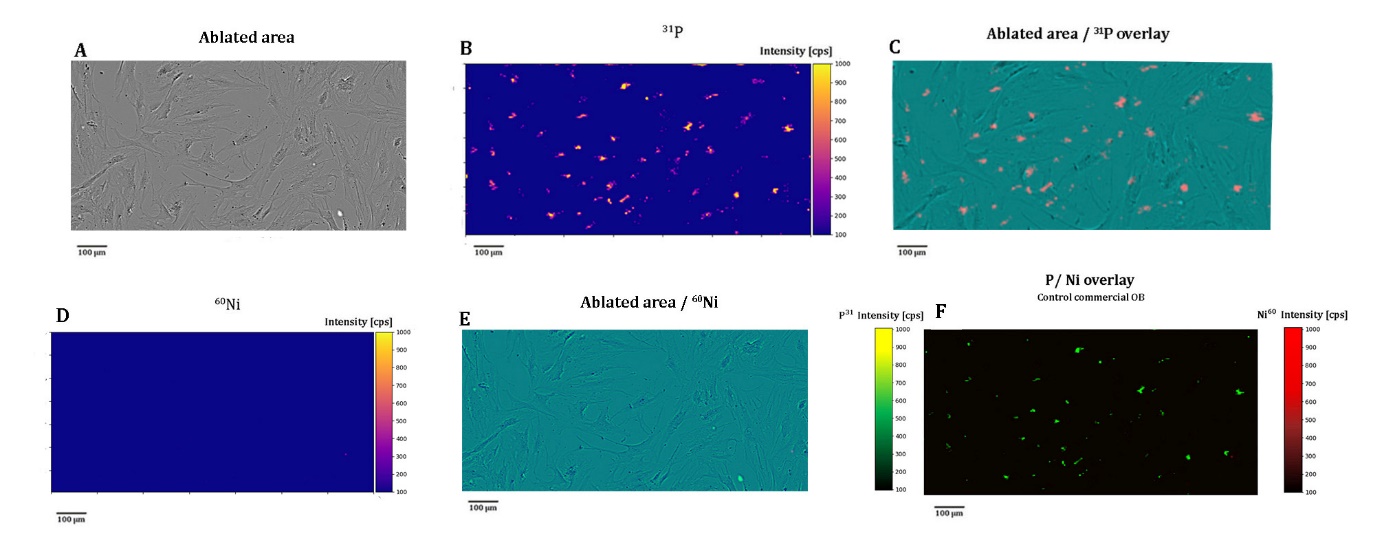

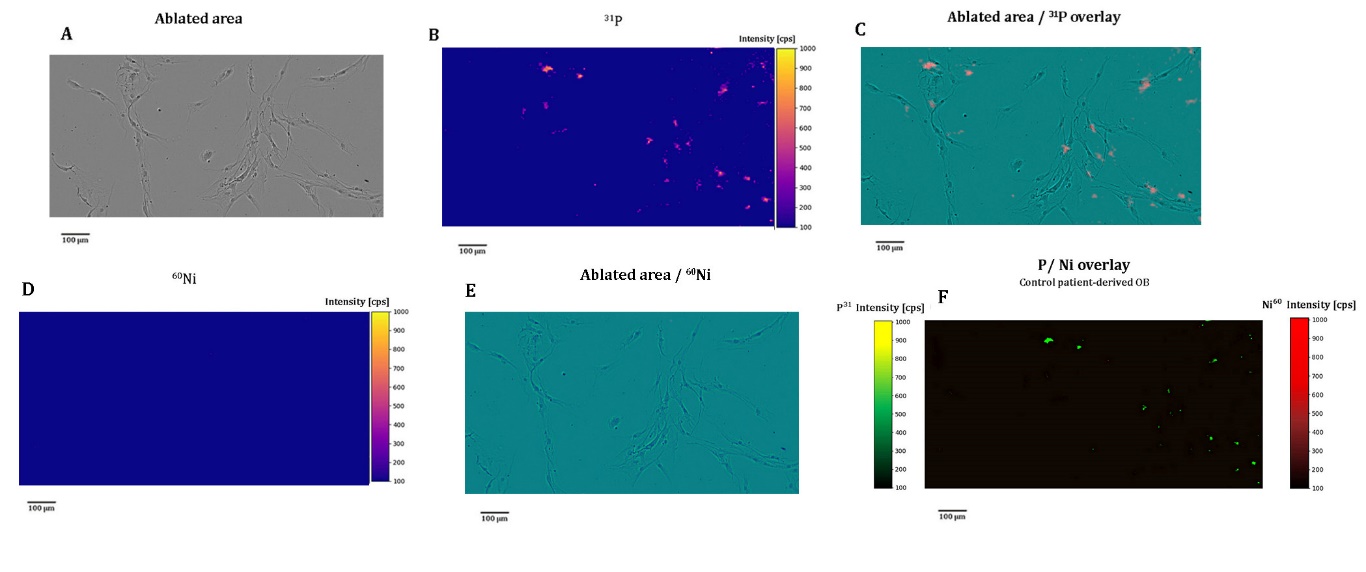


**a**

**b**
